# Retrospective Study on Orthodontic Gingival Recession Correction Using Clear Aligners

**DOI:** 10.3390/medicina60122024

**Published:** 2024-12-08

**Authors:** Sorana Maria Bucur, Eugen Bud, Mioara Decusară, Cristian-Doru Olteanu

**Affiliations:** 1 Department of Dentistry, Faculty of Medicine, “Dimitrie Cantemir” University of Târgu Mureș, 3-5 Bodoni Sandor Str., 540545 Târgu-Mureș, Romania; bucursoranamaria@gmail.com; 2 Department of Orthodontics, Faculty of Dental Medicine, “George Emil Palade” University of Medicine and Pharmacy, Sciences and Technology, 540139 Târgu-Mureș, Romania; 3 Department of Dentistry, Faculty of Medicine and Pharmacy, “Dunărea de Jos” University of Galați, 47 Domnească Str., 800008 Galați, Romania; 4 Department of Orthodontics, Faculty of Dental Medicine, “Iuliu Hațieganu” University of Medicine and Pharmacy, 8 Babeș Str., 400012 Cluj-Napoca, Romania; olteanu.cristian@umfcluj.ro

**Keywords:** clear aligners, gingival recessions, Cairo classification, periodontal evaluation

## Abstract

*Background and Objectives*: Our study aims to evaluate the effectiveness of ClearCorrect^®^ clear aligners (The Straumann Group) in improving gingival recession, which developed following prior fixed orthodontic treatment. The specific objectives were to assess changes in recession severity in patients with orthodontic gingival recessions following aligner therapy and to determine whether the initial severity of recession influenced improvement rates across three categories—mild (RT1), moderate (RT2), and severe (RT3)—as defined by the Cairo classification. *Materials and Methods:* Fifty-six patients with gingival recession from previous orthodontic treatment received aligner therapy for seven to twelve months, focusing on 146 recession sites, primarily in the lower anterior region. Recession severity was classified and tracked as RT1, RT2, and RT3. Statistical analyses, including a Chi-square test, assessed improvement rates across these categories. *Results:* Among severe recessions (RT3), 52% showed no improvement, while 48% exhibited some improvement, with 16% improving to RT1. In the moderate group (RT2), 63% improved to RT1, and 16% resolved. The mild group (RT1) showed a 58% reduction in recession depth. Chi-square testing confirmed a statistically significant difference in improvement rates across the three groups (*p* = 1.44 × 10^−7^). *Conclusions:* Clear aligners may contribute to gingival recession improvement, particularly in mild and moderate cases (RT1 and RT2), though severe cases (RT3) show a more limited response.

## 1. Introduction

A gingival recession occurs when the gingival margin moves apically from its normal position, 1–2 mm coronally to the cementoenamel junction (CEJ), exposing the root surface. Recession can affect any tooth surface, but mucogingival surgery primarily targets vestibular recessions [1], commonly on the buccal surfaces of mandibular central incisors and maxillary molars [2]. These lesions are characterized by attachment loss primarily on the buccal surface, typically with a non-periodontal etiology and minimal interproximal attachment loss [2].

Gingival recession is common in adults, increases with age, and occurs across varying oral hygiene standards. Studies show it affects 88% of those aged ≥65 and 50% of those aged 18–64 with at least one affected site [3]. Clinically, it is significant due to root exposure, leading to aesthetic issues (stains), dentinal hypersensitivity, and cervical lesions (carious and non-carious, including abrasions and erosions). Such lesions can alter the root surface, erasing the CEJ or forming concavities. NCCL prevalence and severity increase with age [4]. As life expectancy rises, gum recession and related root damage become more prevalent. The etiology remains unclear, with multiple predisposing factors complicating causative analysis. Orthodontic appliances can predispose to gingival recession by promoting plaque accumulation. Orthodontic therapy may thus serve as both an etiologic and contributing factor to recession [5]. During tooth movement, periodontal tissue must maintain a stable position around the tooth’s cervical area, requiring adequate attached gingiva for periodontal health and controlled movement [6]. Although orthodontic treatment may contribute to the development of recessions, it does not necessarily lead to their formation [5,7,8]. 

Keratinized gingiva should ideally retain or increase its thickness and height during treatment. Recession is not solely caused by orthodontics but results from irritant, traumatic, or bacterial stimuli in susceptible, thin phenotypes [9].

Traumatic brushing is the most common cause of recession due to inappropriate technique or hard bristles, leading to painless abrasion or ulceration in soft tissues and cervical abrasion in hard tissues [10]. A thorough profile analysis helps diagnose cervical abrasion and plan treatment, as it provides details on abrasion depth and CEJ relations [10,11]. Abraded areas appear darker due to dentin exposure, blending the crown and root dentin [12], and are most visible on prominent teeth and curved arch areas.

Improper flossing can cause gum recession by failing to contour the tooth’s convexity, leading to gingival injury at the angular lines [13]. This often results in a narrow, coronal-apical fissure at the gingival margin. Bacterial plaque-induced recessions, distinct from periodontitis-related recessions involving connective tissue loss, occur on buccal surfaces with minimal attachment loss [14]. These cases may benefit from mucogingival surgery. Diagnosis relies on identifying microbial deposits or inflammation around the exposed root, differentiating it from traumatic brushing recessions, which show clean roots and no inflammation.

The gingival recession classification proposed by Cairo et al. [15] is a treatment-oriented classification that predicts root coverage potential by assessing interdental attachment loss (Clinical Attachment Level-CAL). This classification considers anatomical criteria and is focused on therapy by categorizing recessions into the following:

- Recession type 1 (RT1) refers to gingival recession without interproximal attachment loss. The interproximal cementoenamel junction (CEJ) is not clinically detectable in the mesial and distal tooth parts. Complete coverage of the exposed tooth is possible.
- Recession type 2 (RT2) refers to the involvement of the interproximal attachment. The extent of interproximal attachment loss, measured by the distance from the interproximal CEJ to the interproximal depth, is typically less than or equal to the loss of vestibular attachment. This type of recession commonly occurs in malpositioned, rotated, or extruded vestibular teeth. While complete coverage of the exposed tooth may be achieved, the success rates are generally lower than RT1.
- Recession type 3 (RT3) is characterized by gingival recession with a loss of interproximal attachment, making the CEJ visible. The extent of interproximal attachment loss exceeds that of vestibular attachment loss. Achieving complete coverage of the roots is not feasible, but intervening to achieve a partial result is not contraindicated.

In the classification by Cairo, the assessment of the exposed root surface now incorporates a clinical determination of the presence or absence of the CEJ by Class A or B and the presence or absence of cervical abrasion >0.5 mm.

Class 1 Miller recession involves cases where the recession does not reach the mucogingival junction, with the interproximal periodontal tissues remaining intact and no bone loss. Class 2 recession occurs when the lesion reaches or exceeds the mucogingival junction, but the interproximal tissues remain intact and there is no bone loss. Class 3 recession is characterized by loss of bone tissue. Finally, Class 4 recession indicates severe bone loss [16]. Cairo’s classification addresses limitations of Miller’s system, such as ambiguity between Classes I and II and reliance on “bone or soft tissue loss” for diagnosing interdental periodontal destruction [16,17]. Unlike Miller’s outdated root coverage predictions, Cairo’s system uses clinical attachment loss, making it simpler and more reliable. Further studies are needed to compare surgical outcomes between the two systems [18].

Cairo is a classification system designed to assess the potential for root coverage by evaluating interdental CAL. Cairo RT1 (corresponding to Miller’s Class I and II) anticipates complete root coverage. In Cairo RT2 (overlapping with Miller’s Class III), some randomized clinical trials suggest achieving 100% root coverage when employing various root coverage surgical techniques. In Cairo RT3 superimposed on Miller’s Class IV, root coverage is not attainable [19,20].

The primary reason for treating gingival recession is to meet a patient’s aesthetic preferences. Excessive tooth length due to gingival recession becomes noticeable when smiling and sometimes even during speaking [21,22]. Addressing this aesthetic concern involves mucogingival surgery of the root sheath or orthodontic repositioning of a root in the alveolar process [21,22,23]. The orthodontic procedures may also improve the marginal bone level and spontaneous resolution of gingival recession [24,25]. The patient often experiences heightened sensitivity to temperature, particularly cold, in teeth affected by gingival recession. It can hinder the patient’s ability to maintain proper oral hygiene [23,26].

From an orthodontic perspective, it is feasible to address gingival recessions by relocating the tooth within the alveolar envelope using appliances that employ controlled torque movement. Correcting the root position reduces the extent of gingival recession, thereby creating a more favorable surgical site for the mucogingival surgery planned for cases where root coverage is still needed [25,27].

Clinical observations suggest that even thin tissue phenotypes can be maintained or “thickened” with simple orthodontic treatments [24,27]. Aligners have revolutionized orthodontics, allowing expert practitioners to transform digital plans into clinical results [28,29]. This technique achieves functional, stable alignment by balancing dental arches. Its innovation lies in 3D imaging via digital scanning, enabling orthodontists to customize treatment with dedicated software [28,29,30]. A primary goal of aligner treatment is to achieve dental alignment that enhances stability, function, and aesthetics. Once the transverse dimensions and arch shape are optimized, dental compromises and compensatory movements can be assessed, especially in sagittal discrepancies [31]. Periodontal health, gingival recession, crowding relapse, diastemas, and joint pathologies must also be considered [32]. Aligner treatment focuses on ideal root positioning for stability and periodontal health, with careful tooth torque evaluation relative to initial positioning and alveolar bone anatomy [29,30].

Scientific evidence [33] suggests the correction of gingival recession through orthodontic treatment with aligners, so the primary aim of this study was to assess the correction of orthodontically-induced gingival recessions using invisible aligners [33]. The hypotheses were the following: null hypothesis (H_0_) that the improvement rates in RT3, RT2, and RT1 are independent, with no significant difference between them, or alternative hypothesis (H_1_) that there is a significant difference in improvement rates between RT3, RT2, and RT1.

## 2. Materials and Methods

Initially, 119 patients with gingival recessions after fixed orthodontic treatment were evaluated, but only 56 agreed to proceed with the subsequent aligner therapy. Patients were 2 to 9 months after fixed orthodontic treatment, with an average of 6.7 months. They underwent treatment with invisible aligners for periods ranging from 7 months to one year. The aligner system used was ClearCorrect^®^ aligners of The Straumann Group, founded in Round Rock, Texas, USA. ClearCorrect aligners are crafted using ClearQuartz™, a cutting-edge trilayer plastic designed for optimal performance. Combined with Performance Trimline, this material enhances treatment effectiveness. ClearCorrect aligners prioritize patient comfort and transparency without compromising strength and durability. A properly fitting aligner should cover the teeth, fitting snugly and feeling tight initially. It should cover the gingiva by 0–2 millimeters, with adjustments for undercuts, recession, or other factors. The incisal edges should align flush with no gaps, and the aligner should fit securely over the distal surfaces of the molars, if applicable [34].

This study was conducted according to the guidelines of the Declaration of Helsinki and approved by the Ethics Committee of SC Algocalm SRL, Târgu Mureș, Romania, 03/06.05.2023.

Informed consent was obtained from all subjects involved in this study.

Before commencing orthodontic treatment, we removed fixed mandibular lingual retainers in those with this retention type in the lower arch and patients underwent thorough periodontal evaluations. Except for gingival recessions, none of the patients exhibited periodontal issues, radiological signs, or clinically detectable defects indicating a history of periodontitis. Each patient signed an informed consent form to participate in this study.

The first orthodontic examination included the following assessments: facial assessment, occlusal assessment (presence of all teeth, their position, and dental relationships), TMJ assessment, functional assessment of breathing, swallowing, and any muscle problems of the facial and masticatory muscles, and intraoral and extraoral photographic documentation, precision impressions, gypsum cast models, and various radiographs (orthopantomography and profile teleradiography). Based on these data, we analyzed the cases. The patients came to us from private offices where they did not resolve occlusal or aesthetic issues or experienced a relapse shortly after active orthodontic treatment with brackets.

We planned orthodontic treatment with aligners, considering the main concerns, patients’ expectations, and the functional and aesthetic goals likely to be achieved with orthodontic therapy. The primary goal of orthodontic treatment was to enhance aesthetics by addressing crowding and root exposure associated with gingival recessions on the teeth, which were a result of prior orthodontic treatment. The patients were treated using both upper and lower aligners, even if one arch did not have a gingival recession but experienced an occlusal relapse. The patients were required to wear the aligners for at least 22 hours a day, removing them only while eating [34].

Before and after orthodontic treatment, the following variables were measured clinically with a calibrated periodontal probe [35]:

1. Recession depth (RD) from the free gingival margin to the cementoenamel junction (CEJ);
2. If present, loss of vestibular and interdental attachment or probing pocket depth (PPD), where gingival recession measurements were taken by two doctors, who then compared their results for validation, and if their measurements did not match, they repeated the process together.

We scanned the gypsum cast models using the GOM ATOS Capsule (Zeiss™ GmbH, Braunschweig, Germany), an optical precision measurement tool. This machine employs two 12-megapixel CCD cameras and a blue light fringe projector to capture surface details. It utilizes encoded markers for spatial referencing, while stereo camera technology generates an overdetermined system of equations for each measurement. The device can detect reference markers with an accuracy ranging from 3 µm to 5 µm. The output is a 3D mesh generated by converting numerous triangulated points captured by the cameras into polygons. These scans of the reference models benchmarked the gingival recession measurements in this study and were kept as documentary probes.

Gingival recessions before and after orthodontic treatment were classified using the Cairo classification system. The size of the gingival recessions was measured before and after treatment with aligners. The data were collected in an Excel spreadsheet and then compared statistically.

## 3. Results

Of the total investigated patients, 32 were female and 24 were male, with an average age of 28.4 years.

**Figure 1:**
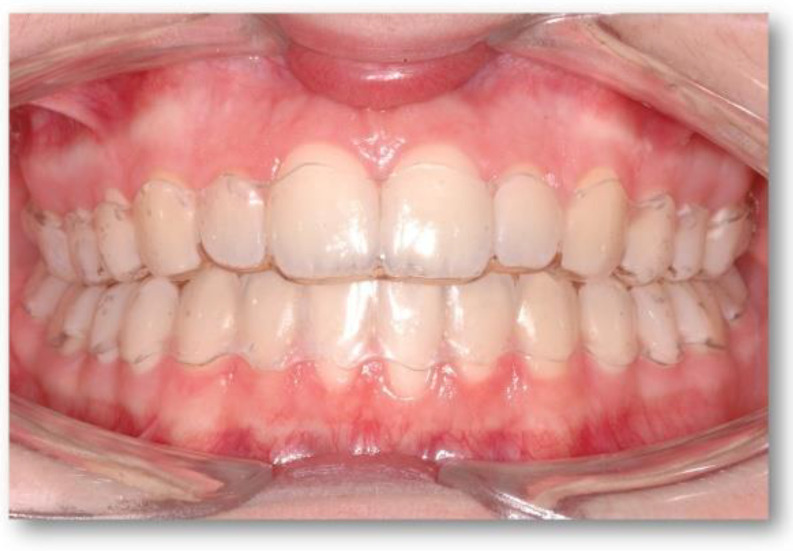
ClearCorrect^®^ aligners in a case with RT1 recessions at 11, 21, 31, 32, 33, and 41.

**Figure 2:**
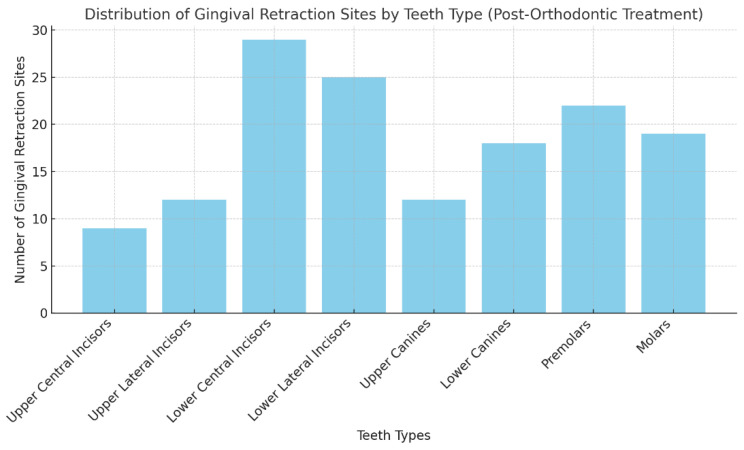
Distribution of gingival recession sites.

Patients exhibited gingival recessions at one, two, or more sites, totaling 146 sites. The distribution of these sites, as illustrated in Figure 1 and Figure 2 was the following:

- 9 in the upper central incisors;
- 12 in the upper lateral incisors;
- 29 in the lower central incisors;
- 25 in the lower lateral incisors;
- 12 in the upper canines;
- 18 in the lower canines;
- 22 in premolars;
- 19 in molars.

Initially, according to the clinical and digital investigations and the Cairo classification, there were 25 locations in RT3, 62 in RT2, and 59 in RT1.

**Figure 3:**
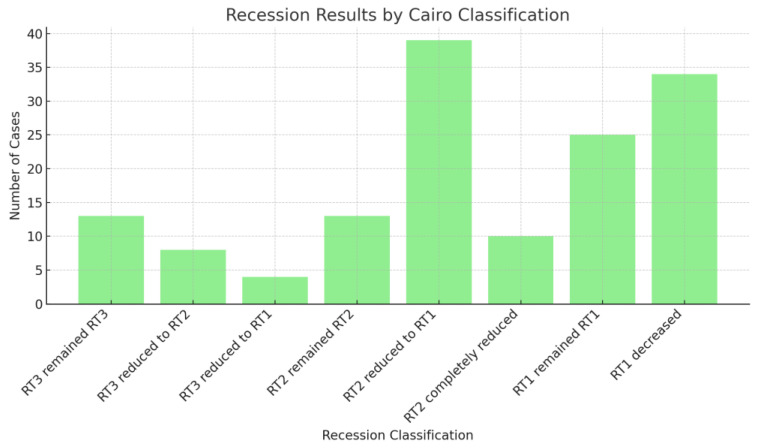
Recession modifications according to Cairo classification.

The study results, illustrated in Figure 3, revealed varying levels of improvement in gingival recessions. Among the locations initially classified as RT3, 13 remained at the RT3 level, indicating no change in this most severe recession category. However, of these RT3 locations, eight improved to RT2, and four further improved to RT1, demonstrating moderate to significant reductions in recession for some cases. For the locations classified as RT2, 13 remained at RT2, while 39 improved to RT1, and 10 experienced complete resolution. This indicates substantial improvement in cases of moderate recession. Among the RT1 locations, 25 showed no change and remained at RT1, while 34 displayed a reduction in recession depth, even within the mild category. Overall, these results emphasize that moderate recession cases (RT2) showed the greatest improvement, with a notable number either fully resolving or decreasing in severity.

To analyze the results effectively, we conducted a comprehensive statistical breakdown using descriptive and inferential statistics. We explored how the recessions in each initial classification (RT3, RT2, and RT1) evolved according to the changes reported to help identify patterns and summarize the progression of cases across the groups. Table 1 contains the descriptive statistics of initial recessions and their changes, and Table 2 shows how many cases in each class were improved after the treatment.

To see if the improvement rates between groups were statistically significant, we applied the Chi-square test. The statistical analysis was performed using Excel and Statistical Package for the Social Sciences (SPSS) software. The current version of IBM SPSS Statistics as of late 2024 is **SPSS Statistics 30.0**, which was released in September 2024. This version includes enhanced features like improved analysis procedures and extended support for statistical workflows, including compatibility with modern platforms.

We calculated the Chi-square statistic and compared it with the critical value for 2 degrees of freedom (since there were three groups). Since we obtained a *p*-value of 1.44 × 10^−7^, less than the typical significance level (0.05), we rejected the null hypothesis (H_0_). It indicated a statistically significant difference in the improvement rates among the RT3, RT2, and RT1 groups. The Chi-square test confirmed that the improvement rates across the groups were not independent and differed significantly.

**Figure 4:**
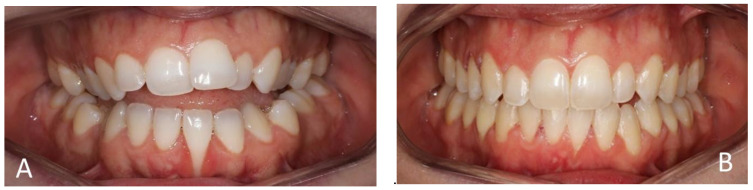
RT2 recession at 31 reduced to RT1. (**A**) Initial frontal aspect; (**B**) Frontal aspect after 10 months of treatment.

**Figure 5:**
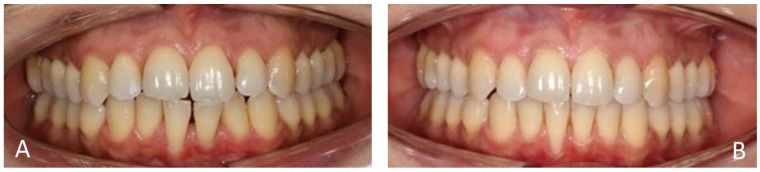
RT2 recession at 41 reduced to RT1. (**A**) Initial frontal aspect; (**B**) Frontal aspect after 7 months of treatment.

**Figure 6:**
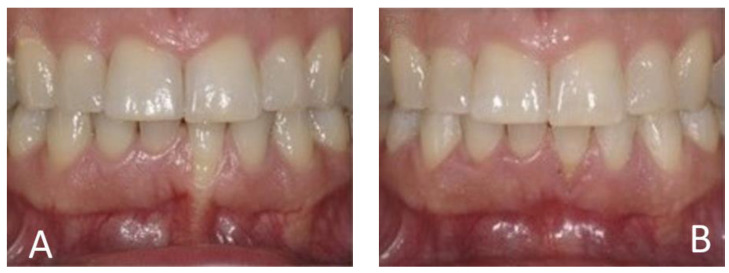
RT3 recession at 31 reduced to RT2. (**A**) Initial frontal aspect; (**B**) Frontal aspect after 10 months of treatment.

**Figure 7:**
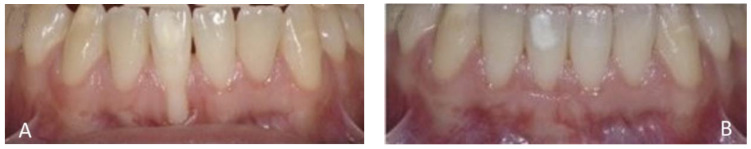
RT3 recession at 31 reduced to RT1. (**A**) Initial frontal aspect; (**B**) Frontal aspect after 10 months of treatment.

Figure 4, Figure 5, Figure 6 and Figure 7 illustrate various recession cases that were successfully treated with orthodontic aligner therapy.

## 4. Discussions

This study’s investigation into gingival recessions before and after orthodontic clear aligner treatment, utilizing the Cairo classification system, provides valuable insights into the relationship between aligner-based orthodontic therapy and gingival health. This research addresses a critical area of concern, as gingival recession can lead to aesthetic and functional problems, such as sensitivity and periodontal disease, which may worsen if not managed properly [26,36]. This study highlights the potential of clear aligners, such as ClearCorrect^®^, to improve gingival recession, especially in mild to moderate cases (RT1 and RT2). The findings suggest that aligners may benefit dental alignment and periodontal health, with moderate recession cases (RT2) showing significant improvement. It underscores the importance of early detection and careful periodontal monitoring in orthodontic patients.

The patients in our group had previously undergone fixed orthodontic treatment, which resulted in gingival recessions at the level of the different teeth as a negative side effect of this type of therapy with fixed attachments. Tooth proclination following orthodontic treatment is significantly associated with an increased risk of gingival recession; initial gingival and bone thickness did not appear to influence the anterior teeth [5,37]. This highlights the importance of thoroughly assessing the risk of recession during diagnosis and treatment planning. Orthodontists should exercise caution when proclining incisors and collaborate with a periodontist to ensure proper periodontal evaluation before and after treatment to minimize recession risk.

The recession pattern in our study is consistent with prior ones, showing that lower incisors are particularly vulnerable to gingival recession during and after orthodontic treatment [37,38]. This could be due to the thin labial bone in this region or the proclination of teeth during treatment, which can induce stress on gingival tissues.

Other authors found that orthodontic treatment can contribute to the onset or worsening of gingival recession, especially in women with limited keratinized tissue [38]. In the mandibular incisor, gingival thickness showed a positive relationship with keratinized tissue width. During orthodontic treatment, teeth might stay within the periodontium limits to prevent complications. A case report [17] presented the long-term healing of gingival recessions on mandibular incisors following orthodontic correction of deep bite malocclusion observed over 30 years. The significant improvement in recession was because of removing direct trauma and orthodontic tooth intrusion, which led to creeping attachment over time. Notably, there was no periodontal intervention before or after the orthodontic procedure.

The upper central and lateral incisors and the upper and lower canines showed some recession but to a lesser extent. Recession was also observed in the premolars (22 sites) and molars (19 sites), indicating that it is not limited to the front teeth and can affect various areas in the dental arch. A case report demonstrated a 3 mm reduction in gingival recession in the incisal, canine, and premolar regions following self-ligating treatment for a Class II Division 1 malocclusion [39]. Improved root coverage was achieved by repositioning the teeth more securely within the alveolar bone and enhancing oral hygiene practices. While it is understood that a single case report cannot be considered definitive evidence, particularly in the context of evidence-based orthodontics, it highlights the responsibility of orthodontists to address more than just the malocclusion, considering the overall periodontal health of the patient.

In our study, for the RT3 group, 52% of sites remained at the same level, while 48% showed improvement, with 16% improving significantly to RT1. In the RT2 group, most cases (nearly 63%) improved to RT1, and a small percentage (16.1%) resolved completely. The RT1 group showed that 57.6% of the sites were positively changed. The analysis provided a clear picture of the progression in each group, allowing for an understanding of the distribution of improvements across different recession types. The RT2 group showed the most notable improvement, with a majority reducing to RT1 or resolving fully.

A study analyzed 220 cases of buccal gingival recession, selected based on specific inclusion and exclusion criteria [18]. Four examiners participated using the following methods: two classified recession defects using Miller’s system, while the other two used Cairo’s system at the start and again after one week. To ensure the reliability of the examiners’ assessments, the intra-class correlation coefficient (ICC) was calculated for 30% of the sample. An ICC value of 0.9 indicated good reliability. Data analysis was performed with Statistical Package for the Social Sciences (SPSS) software, employing Cohen’s kappa and Chi-square tests to evaluate intra- and inter-rater agreement across the two classification methods. Results showed intra-rater consistency for Cairo’s classification at 0.86 and 0.82 and for Miller’s at 0.68. Inter-rater agreement for Cairo’s classification was 0.82 and 0.8, while for Miller’s, it was 0.56 and 0.67. The study concluded that Cairo’s system was more accurate and reliable than Miller’s for assessing gingival recession within the study’s constraints [18].

Before aligner treatment, based on clinical and digital assessments using the Cairo classification, 25 sites were classified as RT3, 62 as RT2, and 59 as RT1.

A cross-sectional study [40] estimated mid-buccal gingival recession (GR) using the 2018 World Workshop Classification System in a population from Northwest Italy. Data from 736 adults in Turin showed that mid-buccal GR ≥ 1 mm affected 57.20% of individuals and 14.56% of teeth. RT1 GR was in 40.90% of patients and 6.29% of teeth, RT2 in 25.82%, and RT3 in 36.68%. RT1 was most frequent in premolars and upper canines, while RT2 and RT3 occurred more in upper molars and lower incisors. RT1 was associated with older age, higher education, and a full-mouth plaque score (FMPS) < 30%, while RT2 and RT3 had older age, lower education, periodontitis, and FMPS > 60%.

Data from 10,676 individuals, representing 143.8 million adults, were analyzed from the National Health and Nutrition Examination Survey 2009–2014 database [41]. GR prevalence, defined as mid-buccal recession ≥1 mm, was classified using the 2018 World Workshop system. Among subjects without periodontitis, 91.6% had GR, dropping to 70.7% in the aesthetic zone. RT1 GR affected 12.4% of patients (whole mouth) and 5.8% in the aesthetic zone, mostly mildly (1–2 mm). RT2 and RT3 GRs had whole-mouth prevalences of 88.8% and 55.0%. Risk factors for RT1 GR included age (35–49), female gender, non-Hispanic White ethnicity, infrequent dental visits, and mandibular incisors [41].

Our study revealed that a significant percentage, 52% of patients with severe RT3-type recession and interdental attachment loss, showed no improvement after treatment. However, some improvements were noted, with eight instances (32%) of RT3 reducing to RT2 and four cases (16%) improving to RT1. The p-value being significantly lower than the common level of 0.05 indicated a statistically significant association between the initial classification of gingival recession (RT level) and the level of improvement. This suggests that the level of initial recession influences the likelihood of improvement in gingival recession severity. While severe recession may persist, some cases can experience improvement in the level of interdental attachment following orthodontic aligner treatment. When combined with periodontal therapy, orthodontics must apply gentle, gradual forces to ensure optimal results, enhancing function and aesthetics and boosting patient confidence.

Another study suggested that clear aligners, combined with periodontal oversight, can effectively treat severe gingival recession and bone defects [33]. This report highlighted the successful use of the Invisalign^®^ system to treat severe gingival recession and bone dehiscence in a young woman through controlled torque, translation, and intrusion movements. Cone-beam computed tomography assessed bone conditions during treatment. The root of a buccally displaced mandibular right central incisor with 9.4 mm bone dehiscence was repositioned into the alveolar bone using Invisalign^®^ and SmartForce^®^ technology. Periodontal monitoring throughout orthodontic treatment showed less gingival recession and bone dehiscence reduced to 3.14 mm, indicating bone regeneration [33]. 

Other authors have different opinions, arguing that the combined treatment of severe periodontitis using fixed appliances results in more reduction in probing pocket depth than aligner treatments [42,43]. Other reviews said there is poor evidence to determine whether clear aligners increase or reduce the risk of gingival recession compared to fixed braces [44]. Aligners may be suitable for orthodontic malocclusions but may not produce as good results for severe cases or when specific tooth movements are required, such as the isolated bodily intrusion of specific elongated teeth [43,45]. Some authors [45] have suggested that orthodontic treatment using aligners may lead to inferior treatment outcomes compared to fixed appliances in adult patients. The available evidence does not advocate for the clinical application of aligners as a treatment modality being as efficacious as the established gold standard of braces [45].

For RT1 recessions without interdental attachment loss, 25 (42%) sites remained unchanged. However, 34 sites (58%) showed a decrease in the size of the recession. This finding is encouraging, as it highlights that early-stage recessions may benefit most from orthodontic treatment, possibly due to improved alignment, reduced plaque build-up, and gingival inflammation, leading to a healthier gingival margin.

These findings indicate that gingival recession may emerge or endure after orthodontic treatment; however, there is a possibility of enhancement in specific cases, particularly those with milder forms of recession (RT1 and RT2). The prevalence of RT3 recessions in numerous instances emphasizes the difficulty of addressing severe recession, especially in the presence of substantial interdental attachment loss. These results underscore the significance of timely detection and intervention in gingival recession, particularly for orthodontic patients, where the meticulous management of periodontal health is paramount. Clear aligners appear to slightly help preserve better periodontal health indicators [37].

The fact that aligner-based treatment can lead to improvements in some types of gingival recession also indicates that such treatment modalities, when combined with appropriate periodontal care, might be less invasive or detrimental to gingival health than traditional fixed appliances, which were frequently associated with recession due to plaque accumulation and difficulties in maintaining oral hygiene [44,46].

Orthodontic treatment can improve tooth positioning and increase buccal periodontal tissue thickness, thereby mitigating the risk of gingival recession in regions with thin buccal bone plates. While orthodontic therapy rarely induces gingival recession, inadequate treatment planning, particularly in areas with compromised bone support, can elevate the risk of recession development. Once gingival recession occurs, periodontal intervention is typically required. However, in cases where V-shaped recession is associated with occlusal trauma, orthodontic realignment may resolve the issue without surgical procedures [47]. There is insufficient evidence to determine whether CA provides superior periodontal health compared to FA during orthodontic treatment [37].

To achieve a more comprehensive understanding of the long-term effects of orthodontic treatment and retention on gingival health [48,49], future research should involve finite element analysis [50,51,52], extend follow-up periods, and explore a broader range of contributing factors. We need to verify the results of our study with more clinical cases. However, the limited financial means of many patients make invisible aligner treatment inaccessible, complicating the enlargement of the study group. Additionally, research comparing different orthodontic treatment types (e.g., fixed appliances versus aligners) could provide more definitive insights into their relative effects on gingival recession.

This study’s strengths were that it addressed the underexplored relationship between clear aligner therapy and gingival health using the Cairo classification system, recognized for its precision in assessing gingival recession, and included data on different types of gingival recession (RT1, RT2, and RT3) and their response to aligner treatment, offering insights into the potential benefits for milder cases (RT1 and RT2). Robust statistical methods, such as ICC values and classification consistency assessments, ensure reliability and validity in data interpretation. This study’s limitations were that a follow-up of 2–9 months may not have captured the long-term effects of aligner treatment on gingival health or potential delayed changes, and it did not consider crucial individual variables like smoking, oral hygiene habits, and genetic predispositions, which are known to influence gingival recession. The limited financial means of patients restricted the inclusion of a bigger and more diverse study group, potentially impacting the generalizability of findings. More extensive, controlled trials are needed to validate our findings.

## 5. Conclusions

This study demonstrated that aligner-based orthodontic treatment may help manage gingival recession and improve milder cases, though severe recessions often remain unchanged. Lower anterior teeth are more susceptible to recession, likely due to a thin labial bone and proclination during previous fixed orthodontic treatment. Proper periodontal evaluation and collaboration between orthodontists and periodontists are essential to minimize recession risks. Cairo classification proved effective for assessing aligner treatment outcomes. While aligner therapy demonstrated advantages over fixed appliances in preserving gingival health, its long-term effects and broader implications require further research.

## Figures and Tables

**Table 1:** Descriptive statistics of initial recessions and their changes.

Initial Classification	Stayed the Same	Improved One Level	Improved Two Levels	Completely Resolved	Further Decreased
RT3 (*n* = 25)	52% (13)	32% (8)	16% (4)	N/A	N/A
RT2 (*n* = 62)	21% (13)	62.9% (39)	N/A	16.1% (10)	N/A
RT1 (*n* = 59)	42.4% (25)	N/A	N/A	N/A	57.6% (34)

**Table 2:** Contingency table for improvement.

Category	Improved	Not Improved	Total
RT3	12	13	25
RT2	49	13	62
RT1	34	25	59

## Data Availability

The original contributions presented in this study are included in the article; further inquiries can be directed to the corresponding authors.
